# The Programmable Nature of Drug-Polymer Systems and Its Implications

**DOI:** 10.3390/polym17060745

**Published:** 2025-03-12

**Authors:** Vlad Ghizdovat, Irina Nica, Lacramioara Ochiuz, Ovidiu Popa, Decebal Vasincu, Dragos Ioan Rusu, Maricel Agop, Ana-Maria Trofin

**Affiliations:** 1 Biophysics and Medical Physics Department, ”Grigore T. Popa” University of Medicine and Pharmacy, 700050 Iași, Romania; vlad.ghizdovat@umfiasi.ro; 2 Department of Odontology-Periodontology, Fixed Prosthesis, “Grigore T. Popa” University of Medicine and Pharmacy, 700115 Iasi, Romania; irina.nica@umfiasi.ro; 3 Department of Pharmaceutical Technology, Faculty of Pharmacy, ”Grigore T. Popa” University of Medicine and Pharmacy, 700050 Iași, Romania; lacramioara.ochiuz@umfiasi.ro; 4 Department of Emergency Medicine, “Grigore T. Popa” University of Medicine and Pharmacy, 700115 Iași, Romania; 5 Faculty of Dental Medicine, “Grigore T. Popa” University of Medicine and Pharmacy, 700050 Iași, Romania; decebal.vasincu@umfiasi.ro; 6 Department of Environmental Engineering, Mechanical Engineering, Faculty of Engineering, “Vasile Alecsandri” University of Bacău, 600114 Bacău, Romania; drusu@ub.ro; 7 Physics Department, “Gheorghe Asachi” Technical University, Bd., No. 59A, 700050 Iași, Romania; 8 Academy of Romanian Scientists, 3 Ilfov, 050044 Bucharest, Romania; 9 General Surgery Department, “Grigore T. Popa” University of Medicine and Pharmacy, 700050 Iași, Romania; ana-maria.trofin@umfiasi.ro

**Keywords:** polymer, drugs, controlled release, multifractality

## Abstract

In our work, we use the multifractal motion theory to apply a multifractal state density conservation law to the polymer-drug release process. This law is specific to the transition from multifractal to nonmultifractal curves corresponding to the polymer-drug release processes. A multifractal diffusion-type law was obtained, which describes the cyclic drug release dynamics until the polymer matrix is fully degraded. The polymer-drug system reveals a programmable nature due to its complex part and scale resolution dependence.

## 1. Introduction

Controlled drug release involves the use of polymeric matrices capable of regulating the diffusion, decomposition, or interaction of drugs with the biological environment. The physicochemical properties of polymers play a central role in this process. These parameters include the molecular weight, crystallinity, hydrophobicity, and porosity of the material. These characteristics can be adjusted to optimize the release kinetics, ensuring a consistent therapeutic profile.

A classic example is biodegradable polymers such as polylactic acid (PLA), polyglycolic acid (PGA), and their copolymers (PLGA). These materials undergo a hydrolysis process in the biological environment, leading to the gradual release of the active substance. Degradation rates can be tuned by changing the ratio of PLA and PGA components, providing flexibility in the treatment design.

Polymers sensitive to external stimuli, such as temperature, pH, or radiation, are another outstanding example. They rely on specific physicochemical properties, such as phase transitions or changes in molecular conformation, to release the drug only in a specific physiological context. For example, thermosensitive polymers, such as those based on poly(N-isopropylacrylamide) (PNIPAAm), can release drugs when the environmental temperature exceeds a critical threshold, which is useful in treating inflammation or tumors.

Innovations in biotechnology and materials engineering have enabled the development of advanced systems for the controlled delivery of drugs to optimize therapeutic efficacy and minimize the adverse effects associated with conventional administration. Polymers represent a versatile class of materials that are widely used in this field due to their tunable properties, biocompatibility, and ability to integrate complex delivery mechanisms. Two key concepts, controlled drug release and programmable polymers, are explored in this context as they contribute to the design of sophisticated, personalized, and efficient systems [1].

The programmable nature of polymers describes the ability of a material to reconfigure its molecular structure or conformation in response to specific stimuli and to return to its initial state when the stimulus disappears. This phenomenon is associated with properties such as shape memory and dynamic covalent bonding.

For example, shape memory polymers that are based on polyurethanes or acrylic polymers are used in implantable devices such as stents or intermittent drug delivery systems. They can be programmed to release the drug according to stimuli, such as body temperature or metabolic variations [2].

The analysis of controlled drug release from polymeric matrices frequently uses mathematical models to better understand the release processes and predict kinetics [3]. In recent decades, several empirical and semi-empirical models have been established to characterize drug release characteristics. Typical instances encompass zero-order and first-order kinetic models, as well as the Higuchi square-root time model, the Hixson–Crowell cube-root model, the Korsmeyer–Peppas power law equation, and the Weibull function [4,5,6]. These models offer efficient methods for fitting in vitro dissolution data and have influenced the design of several drug delivery systems by guiding formulation optimization [7]. Each model is defined by distinct assumptions and fitting parameters; for example, Higuchi’s model produces a linear correlation between drug release and the square root of time, which is suitable for a diffusion-driven release from planar matrices, whereas the Korsmeyer–Peppas model employs a release exponent n to categorize the mechanism (Fickian diffusion, anomalous transport, etc.) based on initial release data [8]. Classical models are often utilized because of their simplicity and historical efficacy in predicting release kinetics.

Despite their prevalence, these traditional models have important limitations when it comes to predicting real-world drug release behavior because they are derived under idealized conditions that are not fully applicable to complex modern formulations. For example, Higuchi’s model—one of the first and most widely used models—assumes a homogeneous matrix with a drug loading beyond the solubility limit, uniform diffusivity, one-dimensional release, and negligible matrix swelling or erosion [8]. In practice, deviating from these conditions (e.g., the drug not being at saturation, significant polymer degradation, and non-planar geometry) causes the Higuchi model to lose accuracy. Similarly, the Korsmeyer–Peppas power law is an empirical equation that is typically limited to the initial ~60% of release, and, therefore, it does not explicitly account for changing the release rates at later times or multi-phase release patterns [8]. More fundamentally, most of the standard models are semi-empirical—they capture the overall release “trend” but do not uniquely correspond to a single physical process. In many cases, multiple phenomena (water penetration, drug diffusion, polymer relaxation, and erosion) occur simultaneously, and a simple equation cannot delineate their individual contributions. Indeed, it has been noted that detailed mechanistic models often reduce to forms equivalent to empirical models when fitted to data, and there is generally “no one-to-one correspondence” between a given empirical fitting equation and a specific physical mechanism [8]. The Weibull model is a good example of an extremely flexible empirical function—it introduces a scale parameter and an exponent that can adapt to different curve shapes (sigmoidal, exponential, etc.), enabling it to fit a wide range of release profiles [8,9]. However, this very flexibility means the Weibull parameters lack direct physical interpretation; a Weibull fit can “approximate and represent a number of different physical situations” without revealing which actual phenomena dominate in a particular system. Consequently, while classical models are useful for initial characterization, their predictive power is limited when the formulation conditions change or when trying to extrapolate from laboratory tests to complex in vivo scenarios. Model parameters often need to be re-fit for each new situation, and the models may fail to capture inflection points, multiphasic release, or the effects of microstructural heterogeneity in the polymer matrix. These limitations have motivated the search for more robust modeling approaches that can bridge the gap between idealized theory and observed drug release kinetics.

One promising approach to improve the realism of drug release models is the application of fractal theory, which accounts for the irregular geometries and heterogeneous dynamics of polymer matrices. Real polymer networks and porous structures can exhibit fractal characteristics (non-integer dimensionality) that influence how a drug diffuses and is released. Incorporating fractal concepts allows the modeling of anomalous diffusion and complex time dependencies that conventional Euclidean models struggle to capture. Recent studies have demonstrated that fractal geometry can quantitatively describe the complex physical changes during drug release. For instance, Yin et al. (2013) analyzed the dissolution of felodipine from osmotic pump tablets using 3D microtomography and found that the tablet’s internal structure evolved in a fractal manner [10]. The fractal dimension of the tablet’s surface (a measure of surface irregularity) increased as the pores enlarged and the matrix eroded, and importantly, this fractal dimension correlated closely with the drug release rate [10]. In their conclusion, the authors noted that the fractal dimension could serve as a “quantitative indicator reflecting the drug release performance”, effectively linking complex morphological changes to release kinetics [10]. Beyond experimental observations, researchers have formulated fractal-based kinetic models to improve release predictions. One such model is the Brouers–Sotolongo fractal kinetic model, which generalizes the classical first-order kinetics into a fractal time domain. This model introduces a fractional exponent (fractal order) that accounts for system heterogeneity and has been shown to fit drug release data with high precision [11]. In fact, fractal kinetic frameworks can unify many of the standard models as special cases—for example, the power law (Korsmeyer–Peppas) and even Higuchi’s law emerge as particular limits of the more general fractal formulations [11]. By adjusting a fractal parameter, the model can mimic various release behaviors, ranging from Fickian diffusion to case-II transport, offering a more flexible yet physically grounded approach. Early applications of these fractal models have been very promising: studies report that incorporating a time- or structure-fractal component yields better fits for experimental release curves and captures subtle release kinetics features that fixed-exponent models miss [11].

In essence, the fractal theory provides a framework to model the inherent complexity of controlled release systems—acknowledging that drug transport may occur in a disordered, multi-scale environment rather than along smooth, ideal pathways.

Taking the above into account, in the present paper, we apply fractal analysis for the drug release process from polymers by making the assumption that the motion of release drug particles takes place on fractured lines as a result of the collisions with other drug particles, as well as the polymeric matrix and release environment. The fractured lines are continuous but nondifferentiable curves, named fractal curves. Further, Nottale’s Scale Relativity Theory [12], or extended scale relativity theory, was developed and applied to different types of drug release systems [13,14,15].

In the dynamics of controlled release systems, as is the case of drug delivery from complex polymer matrices, determinism does not necessarily involve either equable behaviors (periodic movements, auto-structuring, auto-assembly, etc.) or predictability. The theoretical models that describe the evolution of drug-controlled release (DCR) systems were almost exclusively built on linear analysis, where unlimited predictability becomes an automatic quality associated with system dynamics. Further developments in non-linear analysis, together with discoveries on the laws governing chaotic systems, reveal that not only do the mathematical models describing the system dynamics of DCR based on reductionist theory have limited predictability, but also the limitless predictability is rather a natural consequence of their augmentation rather than an attribute of such systems when a description of their dynamics is founded on linear analysis. Hence, non-linearity and chaos reveal common behaviors and manifestations through certain universality of laws that govern the system dynamics of DCR.

Non-linearity and chaoticity are of both structural and functional nature for any DCR system; the interactions between the constitutive entities lead to mutual constraints and implicitly to behaviors of micro-/macroscopic, local/global, and individual/collective types. In such conjecture, the universality of laws for DCR systems becomes natural and must be reflected in mathematical procedures in the form of various theoretical models that could describe their dynamics.

Thus, the success of the above-mentioned models should be understood as gradual/sequential in domains in which differentiability and integrability are still valid. The differentiable and integrable mathematical procedures otherwise suffer when the dynamics of DCR systems must be solved—dynamics that involve both non-linearity and chaoticity. However, dependences related to these procedures remain, becoming necessary to explicitly introduce the scale resolution into the expression of variables associated with DCR dynamics and implicitly into the expression of fundamental equations that govern it. In this context, any variable depends on space and time coordinates (specifically for classical mathematical senses such as differentiability and integrability) along with the scale resolution (e.g., nondifferentiability and non-integrability). In other words, instead of operating with a variable described through a nondifferentiable function, approximations of such dependences obtained by mediation at various scale resolutions will be used. As a result, any variable designed to describe the DCR dynamics will work as the limit of a family of mathematical functions, being nondifferentiable for a null scale resolution and differentiable for non-zero scale resolutions.

This approach in unfolding DCR dynamics obviously involves the development of new geometrical structures along with a new class of models for which the laws of motion, invariant to spatio-temporal transformations, must be integrated into scale laws, invariant to scale resolution transformations. Admitting that such a geometrical framework is one based on the concept of a fractal, the Theory of Scale Relativity in an arbitrary but constant fractal dimension became effective in describing the dynamics of DCR systems [16]. Hence such dynamics could be mathematically described through continuous but nondifferentiable curves (fractal curves), further named “releasing curves”, a situation that occurs when the following consequences are noticeable: (i) any variable designed to describe the dynamics of DCR systems is mathematically expressed through fractal functions, which are functions that depend both on space–time coordinates and scale resolution; (ii) the laws describing the dynamics of DCR systems are invariant both to spatio-temporal transformations and to scale resolution ones; (iii) the dynamics with constraints of a DCR system described by continuous and differentiable curves belonging to an Euclidian space are substituted with dynamics of a same type of system free of any limitation, described by continuous but nondifferentiable curves (fractal curves) from a fractal space.

From this type of perspective, any DCR system treated as a chaotic one that lacks memory has a suspended, dormant history/anthropology and shows a behavior determined by a relatively low number of non-linear interactions. On the opposite, any DCR system treated as a complex one has memory, an active, noticeable history/anthropology, and shows an extremely high number of non-linear interactions. An infinite number of releasing curves could be traced between two points of a fractal space, acting as geodesic of this space. The indiscernibility of these curves results from fractalization through stochasticization due to the stochastic character of releasing content, while their discernibility could be attained through a selection process based on a measurement procedure.

## 2. Mathematical Model

Let us now suppose that a drug-polymer system may be assimilated to a fractal or multifractal mathematical object both in structure and function, given specific conditions. We present several reasons why this can be possible [17,18,19,20]:

- Structural assimilation: Fractals are self-similar entities characterized by the recurrence of analogous patterns at varying sizes. A drug-polymer system, especially in drug delivery or controlled release applications, frequently possesses intricate, hierarchical structures that may display fractal-like characteristics. Polymer networks or hydrogels utilized in drug delivery may exhibit branching or porous patterns that mimic fractals. Microscopic drug release patterns within the system may exhibit a self-similar distribution of the drug across various scales, contingent upon the drug’s diffusion or interaction with the polymer matrix. The aggregation of drug molecules inside a polymer matrix can provide fractal-like patterns, depending on the drug loading technique and release mechanisms employed.
- Functional assimilation: Fractals possess advantageous characteristics, such as scale invariance and self-similarity, which may enhance the functioning of a drug-polymer system. A fractal methodology may be utilized to characterize intricate drug release patterns, including the diffusion of the drug from a polymer matrix or its progressive degradation over time. Fractals, due to their scaling properties, may elucidate the relationship between drug release and its temporal or concentration-dependent dynamics. Furthermore, drug-polymer systems frequently demonstrate non-linear release kinetics or intricate behaviors (e.g., a burst release succeeded by controlled release), which may be modeled utilizing fractal or multifractal mathematics to encapsulate the complex dynamics of drug interaction with the polymer and its environment.
- Fractal geometry in drug-polymer systems: Fractal geometry can be utilized to comprehend the pore architecture of a polymeric drug delivery system since the formation of the polymer network may result in a fractal-like arrangement of the polymer chains. The surface area and permeability of the drug-polymer system may be examined using fractals, as these systems frequently exhibit scaling correlations between the surface area and mass or diffusion characteristics.

In this context, the dynamics of any polymer-drug system can be, by employing the Multifractal Theory of Motion [16], described by continuous and nondifferential curves (multifractal curves). This implies the functionality of the scale covariant derivative.

(1) $$\frac{\hat{d}}{dt}=\partial t+{\hat{V}}^{e}\partial_{e}+D^{ie}\partial_{i}\partial_{e},\;\;i,e=1,2,3$$

where we used the following notations:

(2a) $$\partial_{t}=\frac{\partial }{\partial t},\;\;\partial_{e}=\frac{\partial }{\partial x^{e}},\;\;\partial_{i}\partial_{e}=\frac{\partial }{\partial x^{i}}\frac{\partial }{\partial x^{e}}$$

(2b) $${\hat{V}}^{e}=V_{D}^{e}-iV_{F}^{e},\;\;i=\sqrt{-1}$$

(2c) $$D^{ie}={\frac{1}{4}\left(dt\right)}^{\left[\frac{2}{f\left(\alpha \right)}\right]-1}\left(d^{ie}+i{\bar{d}}^{ie}\right)$$

(2d) $$d^{ie}=\left(\lambda_{+}^{i}\lambda_{+}^{e}-\lambda_{-}^{i}\lambda_{-}^{e}\right),\;\;{\bar{d}}^{ie}=(\lambda_{+}^{i}\lambda_{+}^{e}+\lambda_{-}^{i}\lambda_{-}^{e})$$

In (1) and (2a)–(2d), the quantities have the following meanings [19,20]:

- $x^{e}$ defines the spatial coordinates, expressed through continuous and nondifferentiable mathematical functions that are dependent on scale resolution;
- The temporal coordinate *t* is expressed through continuous and differentiable mathematical functions not dependent on scale resolution;
- $V_{D}^{e}$ defines the differentiable velocity (i.e., the velocity at a differential scale resolution);
- $V_{F}^{e}$ illustrates the nondifferentiable velocity (i.e., the velocity at a nondifferentiable scale resolution);
- $\lambda_{+}^{e}$ and $\lambda_{-}^{e}$ are particular constants of the differentiable–nondifferentiable scale transition coupled with the forward and backward drug-polymer system dynamics, respectively;
- The singularity spectrum of order α, identified through $f\left(\alpha \right)$, is defined by $\alpha =\alpha \left(D_{F}\right)$, where $D_{F}$ represents the fractal dimension of the motion curves of the structural units of any drug-polymer system.

The explicit form of the tensor $D^{ie}$, i.e., that associated with the multifractal scale transition is dictated by multifractalization through stochasticity. Therefore, we can distinguish the following types of polymer-drug system dynamics:

(i) Multifractal polymer-drug system dynamics via Markov stochasticity imposed by the constraints [12,16,21]:

(3) $$\lambda_{+}^{i}\lambda_{+}^{e}=\lambda_{-}^{i}\lambda_{-}^{e}=-2\lambda \delta^{ie}$$

where λ is a diffusion-type coefficient associated with the multifractal–nonmultifractal scale transition and $\delta^{ie}$ is Kronecker’s pseudotensor. In such an alternative:

(4) $$D^{ie}\to i\lambda {\left(dt\right)}^{\left[\frac{2}{f\left(\alpha \right)}\right]-1}$$

So that Equation (1) becomes:

(5) $$\frac{\hat{d}}{dt}=\partial_{t}+{\hat{V}}^{e}\partial_{e}-i\lambda {\left(dt\right)}^{\left[\frac{2}{f\left(\alpha \right)}\right]-1}\partial^{e}\partial_{e}$$

(ii) Multifractal polymer-drug system dynamics by non-Markov stochasticity imposed by the constraints [12,16]:

(6) $$d^{ie}=4\alpha^{\delta^{ie}},\;\;{\bar{d}}^{ie}=-4\beta^{\delta^{ie}}$$

where α and β are the diffusion-type coefficients associated with the multifractal–nonmultifractal scale transition. In such an alternative:

(7) $$D^{ie}=\left(\alpha -i\beta \right){\left(dt\right)}^{\left[\frac{2}{f\left(\alpha \right)}\right]-1}$$

Now, Equation (1) becomes:

(8) $$\frac{\hat{d}}{dt}=\partial_{t}+{\hat{V}}^{e}\partial_{e}-(\alpha -i\beta )\;{\left(dt\right)}^{\left[\frac{2}{f\left(\alpha \right)}\right]-1}\partial^{e}\partial_{e}$$

Assuming now the functionality of the principle of scale covariance, according to which the laws of physics describing polymer-drug system dynamics remain invariant with respect to both spatial and temporal transformations and scale transformations, different conservation laws can be constructed.

For example, if one applies the complex operator (8) to the density of states ρ, then the conservation law takes the form of:

(9) $$\frac{\hat{d}\rho }{dt}=\partial_{t}\rho +{\hat{V}}^{e}\partial_{e}\rho -\left(\alpha -i\beta \right){\left(dt\right)}^{\left[\frac{2}{f\left(\alpha \right)}\right]-1}\partial^{e}\partial_{e}\rho =0$$

or yet explained on resolution scales

(10) $$\partial_{t}\rho +V_{D}^{e}\partial_{e}\rho +\alpha \;{\left(dt\right)}^{\left[\frac{2}{f\left(\alpha \right)}\right]-1}\partial^{e}\partial_{e}\rho =0$$

at differential scale resolutions, i.e.,

(11) $$-V_{F}^{e}\partial \rho -\beta \;{\left(dt\right)}^{\left[\frac{2}{f\left(\alpha \right)}\right]-1}\partial^{e}\partial_{e}\rho =0$$

at nondifferential scale resolutions. Next, if we denote with

(12) $$V^{e}=V_{D}^{e}-V_{F}^{e}$$

the velocity associated with the multifractal–nonmultifractal scale transition, by adding Equations (10) and (11), the conservation law of the density of states associated with the multifractal–nonmultifractal scale transition is obtained as:

(13) $$\partial_{t}\rho +V^{e}\partial_{e}\rho +(\alpha -\beta ){\left(dt\right)}^{\left[\frac{2}{f\left(\alpha \right)}\right]-1}\partial^{e}\partial_{e}\rho =0$$

With respect to Equation (9), let us note the following:

(i) The rate of change in the density field, multifractal convection, and multifractal dissipation of the same field are in equilibrium at any time;
(ii) The presence of the complex dissipative coefficient $\left(\alpha -i\beta \right){\left(dt\right)}^{\left[\frac{2}{f\left(\alpha \right)}\right]-1}$ specifies that the polymer-drug system exhibits a programmable nature, in agreement with [22,23]. In such a context, after each release process, the polymer-drug system repeats the same cycle at a different resolution scale until the drug concentration reaches zero and the polymer network starts to degrade;
(iii) In our opinion, the non-linear behavior of the polymer-drug system, responsible for the programmable nature mentioned above, is explicated not only by its complex part but also by its scale resolution dependence ${\left(dt\right)}^{\left[\frac{2}{f\left(\alpha \right)}\right]-1}$. In this way, the presence of the product between $\left(\alpha -i\beta \right)$ and ${\left(dt\right)}^{\left[\frac{2}{f\left(\alpha \right)}\right]-1}$ also specifies the sense in which release dynamics take place, according to (ii).

In the following, we present the consequences of such a differential equation in modeling polymer-drug system dynamics. For this purpose, let us consider the differential Equation (13) in the one-dimensional case and, which is subject to the following constraint

(14) $$V^{e}\equiv (0,0,V=const.)$$

This becomes

(15) $$\frac{\partial \rho }{\partial t}+V\frac{\partial \rho }{\partial z}-D\frac{\partial^{2}\rho }{\partial z^{2}}=0$$

where we made the notation:

(16) $$D=(\beta -\alpha )\;{\left(dt\right)}^{\left[\frac{2}{f\left(\alpha \right)}\right]-1}$$

where *D* depends on the physical processes occurring in the drug release from polymers and also on a scale resolution and its properties.

The above model can describe, for example, at various scale resolutions, non-linear behaviors of polymer-drug systems that simultaneously obey release and diffusion processes through transitions from turbulence to laminar dynamics. From this perspective, the turbulence–laminar transition will work as a multifractal–nonmultifractal scale transition where *V* denotes the drug release rate associated with such a transition.

By making the variable change

(17) $$\rho \left(z,t\right)=n\left(z,t\right)exp(\frac{Vz}{2D}-\frac{V^{2}t}{4D})$$

which implies:

(18) $$\frac{\partial \rho }{\partial t}\;=\;(\frac{\partial n}{\partial t}-\frac{V^{2}n}{4D})exp(\frac{Vz}{2D}-\frac{V^{2}t}{4D})\;\frac{\partial \rho }{\partial z}\;=\;(\frac{\partial n}{\partial z}+\frac{Vn}{2D})exp(\frac{Vz}{2D}-\frac{V^{2}t}{4D})\;\frac{\partial^{2}\rho }{\partial z^{2}}\;=\;(\frac{\partial^{2}n}{\partial z^{2}}+\frac{V}{D}\frac{\partial n}{\partial z}+\frac{V^{2}n}{4D})exp(\frac{Vz}{2D}-\frac{V^{2}t}{4D})$$

the differential Equation (15) becomes:

(19) $$\frac{\partial n}{\partial t}=D\frac{\partial^{2}n}{\partial z^{2}}$$

From the initial and boundary conditions

(20) $$\rho \left(z,0\right)=\rho_{0}=const\;\rho \left(0,t\right)=0$$

the boundary conditions corresponding to *n* are obtained, i.e.,

(21) $$n\left(z,0\right)=n_{0}exp(\frac{Vz}{2D})\;n\left(0,t\right)=0$$

This problem can be reduced to one without boundary conditions by extending the solution to include the region z<0, where *n* assumes an initial distribution that simulates the boundary condition for z=0 at any time t>0. At the same time, one can choose

(22) $$n\left(z,0\right)=n(-z,0)$$

so that for any extended region, the initial condition will be

(23) $$n\left(z,0\right)=\left\{\begin{array}{c} n_{0}\exp\left(\frac{Vz}{2D}\right),\;z<0\\ -n_{0}\exp\left(-\frac{Vz}{2D}\right),\;z>0 \end{array}\right.$$

The problem is how to solve the diffusion equation for an initially specified dimensional distribution in an unbounded medium. This can be solved by developing the solution as a Fourier integral:

(24) $$n\left(z,t\right)=\int_{-\infty }^{+\infty }\phi \left(k,t\right)\exp\left(ikz\right)dk$$

(25) $$\phi \left(k,t\right)=\frac{1}{2\pi }\int_{-\infty }^{+\infty }n(z^{\prime },t)\exp\left(-ikz^{\prime }\right)dz\prime$$

After substituting the differential Equation (19), we obtain

(26) $$\frac{\partial \phi }{\partial t}+k^{2}D\phi =0$$

with the solution:

(27) $$\phi \left(k,t\right)=\phi \left(k,0\right)exp(-k^{2}Dt)$$

where $\phi \left(k,0\right)$ was obtained by substituting the initial distribution *n* into Equation (25). Therefore, Equation (24) can be expressed as an integral over the entire initial distribution as follows:

(28) $$n\left(z,t\right)=\frac{1}{2\pi }\int_{-\infty }^{+\infty }\int_{-\infty }^{+\infty }n(z^{\prime },0)\exp\left(-k^{2}Dt\right)exp\left[ik(z-z^{\prime })\right]dz\prime dk$$

Once the integral above is resolved with respect to *k*, solving it for *n* becomes straightforward, leading to:

(29) $$n\left(z,t\right)=\frac{1}{{\left(4\pi Dt\right)}^{\frac{1}{2}}}\int_{-\infty }^{+\infty }n(z^{\prime },0)\exp\left[-\frac{{\left(z-z^{\prime }\right)}^{2}}{4Dt}\right]dz\prime$$

The solution can be completed by substituting Equation (23) into Equation (29) and calculating the integral. The result for $\rho \left(z,t\right)$ is given by equation:

(30) $$\rho \left(z,t\right)=\frac{n_{0}}{2}\exp\left(-\frac{Vt}{D}\right)+\frac{n_{0}}{2}\exp\left(-\frac{Vt}{D}\right)\cdot \left[\frac{erf(z-Vt)}{{\left(4Dt\right)}^{\frac{1}{2}}}+\frac{erf(z+Vt)}{{\left(4Dt\right)}^{\frac{1}{2}}}\right]$$

where $\mathrm{erf}\left(x\right)$ is the Laplace function given by

(31) $$\mathrm{erf}\left(x\right)=\frac{1}{2\sqrt{\pi }}\int_{0}^{\infty }\exp\left(-x^{2}\right)dx$$

values of this function being tabulated.

The drug release rate from the polymer at z=0 is given by:

(32) $$\begin{array}{cc} I\left(t\right)=-\vec{j}\left(0,t\right){\hat{e}}_{z} & =D{\left(\frac{\partial n}{\partial t}\right)}_{z=0}\\ & =\rho_{0}\left\{\frac{V}{2}\cdot \left[1+\mathrm{erf}{\left(\frac{V^{2}t}{4D}\right)}^{\frac{1}{2}}+{\left(\frac{D}{\rho t}\right)}^{\frac{1}{2}}exp\left(-\frac{V^{2}t}{4D}\right)\right]\right\} \end{array}$$

**Figure 1:**
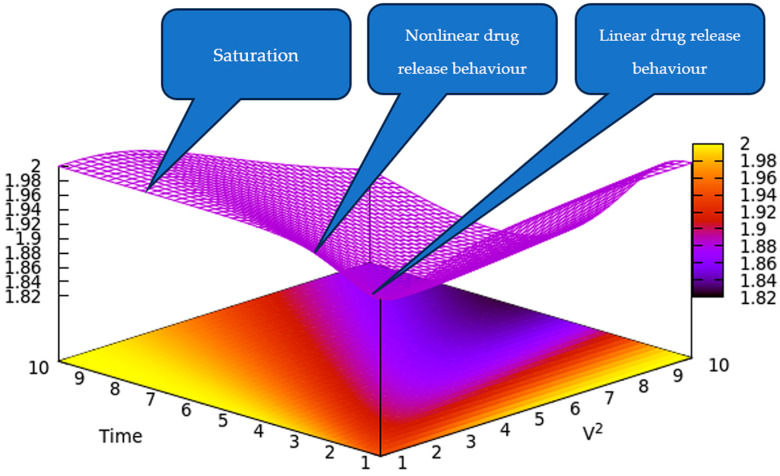
A 3D representation of the drug release rate is indicated by the color gradient as a function of time and *V*^2^ according to Equation (32). *V*^2^ and time are reduced coordinates.

Figure 1 illustrates a 3D depiction of the drug release rate in relation to time and *V*^2^. This provides a qualitative depiction of the drug release rate, indicating that an increase in quadratic speed could result in the highest release rate over a shorter time period. However, over an extended period, the drug release rate may become saturated.

In this way, we have obtained, through our model, a typical controlled drug release from a polymeric matrices release curve, in agreement with the experimental data [24,25,26].

According to Equation (32), the diffusion current $I\left(t\right)$ is infinite at t=0. This is because an infinite gradient of the concentration has been artificially assumed at z=0, t=0.

For $t\ll t_{e}=\frac{4D}{V^{2}}$, Equation (32) becomes

(33) $$I\left(t\right)=n_{0}\left[{\left(\frac{D}{\pi t}\right)}^{\frac{1}{2}}+\frac{V}{2}\right]$$

showing that the drug release rate is controlled both by the drug release rate and diffusion velocity. We must mention that $t_{e}=\frac{4D}{V^{2}}$ is a critical time in the drug-polymer dynamics (a drug release time).

## 3. Temporal Drug Release Patterns

Equation (19) remains invariant with respect to the special transformation group [16,27]:

(34) $$z=\frac{z\prime }{\gamma t+\delta },\;\;t\prime =\frac{\alpha t+\beta }{\gamma t+\delta }$$

In such a context, let the second Equation (34) be reconsidered, which represents the homographic action of the generic matrix:

(35) $$\hat{M}=\left(\begin{array}{cc} \alpha & \beta \\ \gamma & \delta \end{array}\right)$$

The issue that needs to be tackled is the following: a relation must be found between the ensemble of matrices $\hat{M}$ and the ensemble of values pertaining to t, for which $t^{\prime }$ remains constant.

From a geometrical point of view, this means finding the ensemble of points $\left(\alpha ,\beta ,\gamma ,\delta \right)$, which univocally correspond to the values of the parameter t. By using the second Equation (34), the issue is solved by a Riccati differential equation, which can be obtained as a consequence of the constancy of $t^{\prime }$: $dt^{\prime }=0$.

(36) $$dt+\omega_{1}t^{2}+\omega_{2}t+\omega_{3}=0$$

where the following notations are used:

(37) $$\omega_{1}=\frac{\gamma d\alpha -\alpha d\gamma }{\alpha \delta -\beta \gamma },\;\omega_{2}=\frac{\delta d\alpha -\alpha d\delta +\gamma d\beta -\beta d\gamma }{\alpha \delta -\beta \gamma },\;\omega_{3}=\frac{\delta d\beta -\beta d\delta }{\alpha \delta -\beta \gamma }$$

It is then easily noticeable that the metric

(38) $$ds^{2}=\frac{{\left(\alpha d\delta +\delta d\alpha -\beta d\gamma -\gamma d\beta \right)}^{2}}{4{\left(\alpha \delta -\beta \gamma \right)}^{2}}-\frac{d\alpha d\delta -d\beta d\gamma }{\alpha \delta -\beta \gamma }$$

is in a direct relation with the discriminant of the quadratic polynomial from Equation (36)

(39) $$ds^{2}=\frac{1}{4}\left({\omega_{2}}^{2}-4\omega_{1}\omega_{2}\right)$$

The three differential forms from Equation (37) constitute a coframe [16,27] at any point of the absolute space. This allows the translation of the geometric properties of the absolute space to the algebraic properties linked to the differential Equation (36).

The simplest of these properties refer to the dynamics on matrix geodesics, which are directly translated to statistical properties. In this case, the 1-forms $\omega_{1},\;\omega_{2},\;\omega_{3}$ are differentiated exactly in the same parameter, the length τ of the geodesic arc. Along these geodesics, Equation (36) is transformed into a Riccati-type differential equation:

(40) $$\frac{dt}{d\tau }=P\left(t\right),\;\;P\left(t\right)=a_{1}t^{2}+2a_{2}t+a_{3}$$

Here, the parameters $a_{1}$, $a_{2}$, $a_{3}$ are constants that characterize a certain geodesic of the family.

For obvious reasons it is important to identify the most general solution of the differential Equation (40). Reference [28] offers a modern and pertinent method for the integrability of the Riccati differential equation. For the present work, it is sufficient to note that the relations:

(41) $$t_{0}=-\frac{a_{2}}{a_{1}}+\frac{i}{a_{1}}\Omega ,\;\;{\bar{t}}_{0}=-\frac{a_{2}}{a_{1}}-\frac{i}{a_{1}}\Omega ,\;\;\Omega^{2}=a_{3}a_{1}-{a_{2}}^{2},\;\;i=\sqrt{-1}$$

can be assimilated to the roots of the polynomial $P\left(t\right)$. As such, the homographic transformation needs to be performed first:

(42) $$z=\frac{t-t_{0}}{t-{\bar{t}}_{0}}$$

and now it is easy to see, by means of direct calculation, that z is a solution of the differential equation:

(43) $$\dot{z}=2i\Omega z,\;\;z\left(\tau \right)=z\left(0\right)e^{i\Omega \tau }$$

Therefore, if the initial condition $z\left(0\right)$ is conveniently expressed, it is possible to obtain the general solution of the differential Equation (40) by inversing the transformation (42), which implies:

(44) $$t=\frac{t_{0}+rexp\left[2i\Omega \left(\tau -\tau_{0}\right)\right]{\bar{t}}_{0}}{1+rexp\left[2i\Omega \left(\tau -\tau_{0}\right)\right]}\;$$

where r and $r_{0}$ are two real constants specific to the solution.

(45) $$t=\frac{t_{0}+rexp\left[2i\Omega \left(\tau -\tau_{0}\right)\right]{\bar{t}}_{0}}{1+rexp\left[2i\Omega \left(\tau -\tau_{0}\right)\right]}\;$$

By using the relations (41), it is possible to obtain the solution in real terms, i.e.,:

(46) $$z=-\frac{a_{2}}{a_{1}}+\frac{\Omega }{a_{1}}\left[\frac{2r\sin\left[2\Omega \left(\tau -\tau_{0}\right)\right]}{1+r^{2}+2r\cos\left[2\Omega \left(\tau -\tau_{0}\right)\right]}+i\frac{1-r^{2}}{1+r^{2}+2r\cos\left[2\Omega \left(\tau -\tau_{0}\right)\right]}\right]\;$$

which highlights a self-modulation of Ω through the Stoler-type transformation [29], which leads to a complex form of this complex parameter. Moreover, if it is noted:

(47) r=coth⁡s 

Equation (46) becomes:

(48) $$z=-\frac{a_{2}}{a_{1}}+\frac{\Omega }{a_{1}}h\;$$

where h has the expression

(49) $$h=-i\frac{\cosh s-exp\left[-2i\Omega \left(\tau -\tau_{n}\right)\right]\sinh s}{\cosh s+exp\left[-2i\Omega \left(\tau -\tau_{n}\right)\right]\sinh s}\;$$

In accordance with [16,27], this complex parameter operates as a harmonic mapping between the usual space (i.e., the measurement space) and the hyperbolic one.

**Figure 2:**
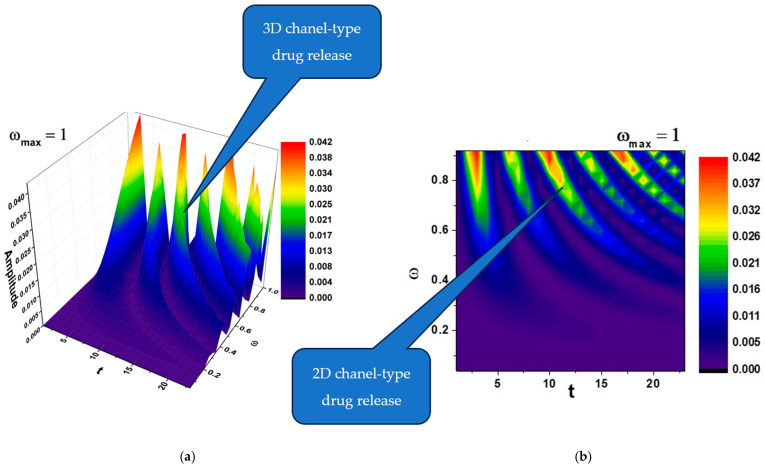
(**a**,**b**) The 3D and contour plots of the solution $Re\left(a_{1}z+a_{2}\right)\equiv Amplitude$ for the maximum value of the pulsation-type characteristic $\omega_{max}=1$ where Ω≡ω and t≡τ.

**Figure 3:**
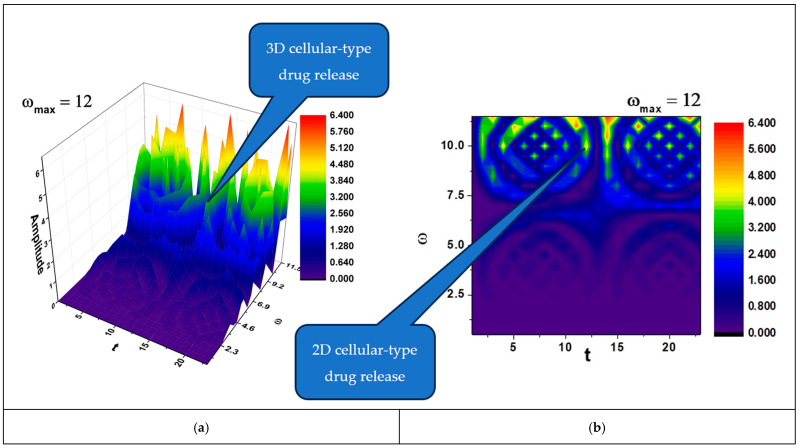
(**a**,**b**) The 3D and contour plots of the solution $Re\left(a_{1}z+a_{2}\right)\equiv Amplitude$ for the maximum value of the pulsation-type characteristic $\omega_{max}=12$ where Ω≡ω and t≡τ.

In Figure 2a,b and Figure 3a,b, self-modulations of the drug release rate in forms that can “mimic” channel-type and cellular-type self-structures are presented.

According to the literature, channel-type structures, like the ones that can be seen in Figure 2a,b, are often used for sustained release formulations, allowing gradual drug diffusion over time [30].

Also, according to the literature, cellular-type structures, like the ones that can be seen in Figure 3a,b, are preferred for targeted delivery [31], where the drug is released in response to specific biological conditions (e.g., tumor microenvironment).

## 4. Hysteretic-Type Behaviors in a Drug-Polymer System

Applying the multifractal operator (5) in the vectorial form to the internal energy per unit volume, ρε, and adopting the principle of scale covariance [12,16], we obtain the internal energy per unit volume conservation law:

(50) $$\frac{\hat{d}\left(\rho \varepsilon \right)}{dt}=\frac{\partial \left(\rho \varepsilon \right)}{\partial t}+V^{l}\partial_{l}\left(\rho \varepsilon \right)-i\lambda {\left(dt\right)}^{\left[\frac{2}{f\left(\alpha \right)}\right]-1}\partial^{l}\partial_{l}\left(\rho \varepsilon \right)=0$$

For the types of movements mentioned above, separating the real part from the imaginary one in Equation (50), we obtain:

(51) $$\frac{\partial (\rho \varepsilon )}{\partial t}+\partial_{l}(\rho \varepsilon V_{D}^{l})=(\rho \varepsilon )\partial_{l}V_{D}^{l}$$

(52) $$V_{F}^{l}\partial_{l}(\rho \varepsilon )=-{\lambda^{2}\left(dt\right)}^{\left[\frac{2}{f\left(\alpha \right)}\right]-1}\partial^{l}\partial_{l}\left(\rho \varepsilon \right)$$

One can notice that, although there is internal energy per unit volume transport at a differentiable scale, a similar phenomenon (convection transport) at a fractal scale occurs.

Let us now consider the Madelung scenario for describing polymer-drug dynamics, given by the differential equations set from multifractal hydrodynamics [16,27]:

(53) $$\partial_{t}V_{D}^{i}+\left(V_{D}^{l}\partial_{l}\right)V_{D}^{i}=-\partial^{i}Q$$

(54) $$\partial_{t}\rho +\partial^{i}\left(\rho V_{D}^{i}\right)=0$$

where

(55) $$Q=-2{\lambda^{2}\left(dt\right)}^{\left[\frac{4}{f\left(\alpha \right)}-2\right]}\frac{\partial^{l}\partial_{l}\sqrt{\rho }}{\sqrt{\rho }}=-\frac{V_{F}^{l}V_{Fl}}{2}-{\lambda \left(dt\right)}^{\left[\frac{2}{f\left(\alpha \right)}-1\right]}\partial_{i}V_{F}^{i}$$

(56) $$V_{D}^{i}=2\lambda {\left(dt\right)}^{\left[\frac{4}{f\left(\alpha \right)}-2\right]}\partial^{i}\phi ,\;\;V_{F}^{i}={\lambda \left(dt\right)}^{\left[\frac{2}{f\left(\alpha \right)}-1\right]}\partial^{i}\phi$$

(57) $$\psi =\sqrt{\rho }e^{i\phi },\;\;\bar{\psi }=\sqrt{\rho }e^{-i\phi },\;\;\rho =\psi \bar{\psi }$$

where Q is the specific multifractal potential, ψ is the state function, $\sqrt{\rho }$ is the amplitude, and ϕ is the phase of the state function. We note that the specific multifractal potential can be related to the multifractal stress tensor:

(58) $${\hat{\sigma }}_{il}=-2{\lambda^{2}\left(dt\right)}^{\left[\frac{4}{f\left(\alpha \right)}\right]-2}\rho \partial_{i}\partial_{l}ln\rho$$

through relation

(59) $$\partial^{i}{\hat{\sigma }}_{il}+\rho \partial_{l}Q=0$$

We must mention that the polymer-drug complex system can be assimilated, both structurally and functionally, to a complex fluid [19].

In such a context, let us reconsider Equations (51), (53), and (54) in the plane symmetry (*x*,*y*), with constraint (56), and $\hat{\sigma }$ in the diagonal form. Also, let us assume that the variation of $\hat{\sigma }$ is induced by the variations in internal energy per unit volume and state density, $\partial_{l}\sigma =\nu \partial_{l}\left(\rho \varepsilon \right)$, with ν=const. Then, in dimensionless variables,

(60) $$\omega t=\tau ,kx=\xi ,ky=\eta \;\frac{V_{x}k}{\omega }=V_{\xi },\frac{V_{y}k}{\omega }=V_{\eta },\frac{\rho }{\rho_{0}}=N,\;\frac{\varepsilon }{\varepsilon_{0}}=\Theta$$

Equations (51), (53), and (54) become:

(61) $$\frac{\partial }{\partial \tau }\left(NV_{\xi }\right)+\frac{\partial }{\partial \xi }\left(NV_{\xi }^{2}\right)+\frac{\partial }{\partial \eta }\left(NV_{\xi }V_{\eta }\right)=-\frac{\partial \left(N\Theta \right)}{\partial \xi }\;\frac{\partial }{\partial \tau }\left(NV_{\eta }\right)+\frac{\partial }{\partial \xi }\left(NV_{\xi }V_{\eta }\right)+\frac{\partial }{\partial \eta }\left(NV_{\eta }^{2}\right)=-\frac{\partial \left(N\Theta \right)}{\partial \eta }\;\frac{\partial N}{\partial \tau }+\frac{\partial }{\partial \xi }\left(NV_{\xi }\right)+\frac{\partial }{\partial \eta }\left(NV_{\eta }\right)=0\;\frac{\partial (N\Theta )}{\partial \tau }+\frac{\partial }{\partial \xi }\left(N\Theta V_{\xi }\right)+\frac{\partial }{\partial \eta }\left(N\Theta V_{\eta }\right)=N\Theta \left(\frac{\partial V_{\xi }}{\partial \xi }+\frac{\partial V_{\eta }}{\partial \eta }\right)$$

where the functional scaling relation, $\nu k^{2}/\omega^{2}=1$, is considered. In Equations (60) and (61), $\rho_{0}$ corresponds to the density of the complex fluid at equilibrium, $\varepsilon_{0}$ to the energy of the complex fluid at equilibrium, ω to the complex fluid pulsation, and *k* to the inverse of a characteristic length of the complex fluid. For the ideal gas case, ν will be the square of the acoustic characteristic velocity and σ the kinetic pressure.

For numerical integration, we consider the initial conditions:

(62) $$V_{\xi }\left(0,\xi ,\eta \right)=0,\;V_{\eta }\left(0,\xi ,\eta \right)=0\;N\left(0,\xi ,\eta \right)=\frac{1}{4},\;\Theta \left(0,\xi ,\eta \right)=\frac{1}{4}\;0\leq \xi \times \eta \leq 1\times 1$$

and the boundary ones:

(63) $$V_{\xi }\left(\tau ,0,\eta \right)=0,\;\;V_{\xi }\left(\tau ,1,\eta \right)=0\;V_{\eta }\left(\tau ,0,\eta \right)=0,\;\;V_{\eta }\left(\tau ,1,\eta \right)=0\;N\left(\tau ,0,\eta \right)=\frac{1}{4},\;\;N\left(\tau ,1,\eta \right)=\frac{1}{4}\;\Theta \left(\tau ,0,\eta \right)=\frac{1}{4},\;\;\Theta \left(\tau ,1,\eta \right)=\frac{1}{4}\;V_{\xi }\left(\tau ,\xi ,0\right)=0,\;\;V_{\xi }\left(\tau ,\xi ,1\right)=0\;\;\;\;\;\;\;\;\;\;\;\;\;\;\;\;\;\;\;\;\;\;\;\;\;\;\;\;\;\;\;\;\;\;\;\;\;\;V_{\eta }\left(\tau ,\xi ,0\right)=0,\;\;V_{\eta }\left(\tau ,\xi ,1\right)=0\;\;\;\;\;\;\;\;\;\;\;\;\;\;\;\;\;\;\;\;\;\;\;\;\;\;\;\;\;\;\;\;\;\;\;\;\;\;N\left(\tau ,\xi ,0\right)=N_{0}\exp\left[-\frac{{\left(\tau -\frac{1}{4}\right)}^{2}}{(1/4{)}^{2}}\right]\cdot \exp\left[-\frac{{\left(\xi -\frac{1}{2}\right)}^{2}}{(1/4{)}^{2}}\right]\;N\left(\tau ,\xi ,1\right)=\frac{1}{4}\;\;\;\;\;\;\;\;\;\;\;\;\;\;\;\;\;\;\;\;\;\;\;\;\;\;\;\;\;\;\;\;\;\;\;\;\;\;\;\;\;\;\;\;\;\;\;\;\;\;\;\;\;\;\;\;\;\;\;\;\;\;\;\;\;\;\;\;\;\;\Theta \left(\tau ,\xi ,0\right)=\Theta_{0}\exp\left[-\frac{{\left(\tau -\frac{1}{4}\right)}^{2}}{(1/4{)}^{2}}\right]\cdot \exp\left[-\frac{{\left(\xi -\frac{1}{2}\right)}^{2}}{(1/4{)}^{2}}\right],\;\Theta \left(\tau ,\xi ,1\right)=\frac{1}{4}\;\;\;\;\;\;\;\;\;\;\;\;\;\;\;\;\;\;\;\;\;\;\;\;\;\;\;\;\;\;\;\;\;\;\;\;\;\;\;\;\;\;\;\;\;\;\;\;\;\;\;\;\;\;\;\;\;\;\;\;\;\;\;\;\;\;\;\;\;$$

In the boundary condition (63), we assumed that the perturbation has a space–time Gaussian profile, N0 is the maximum normalized state density, and $\Theta_{0}$ is the maximum normalized internal energy per unit volume.

**Figure 4:**
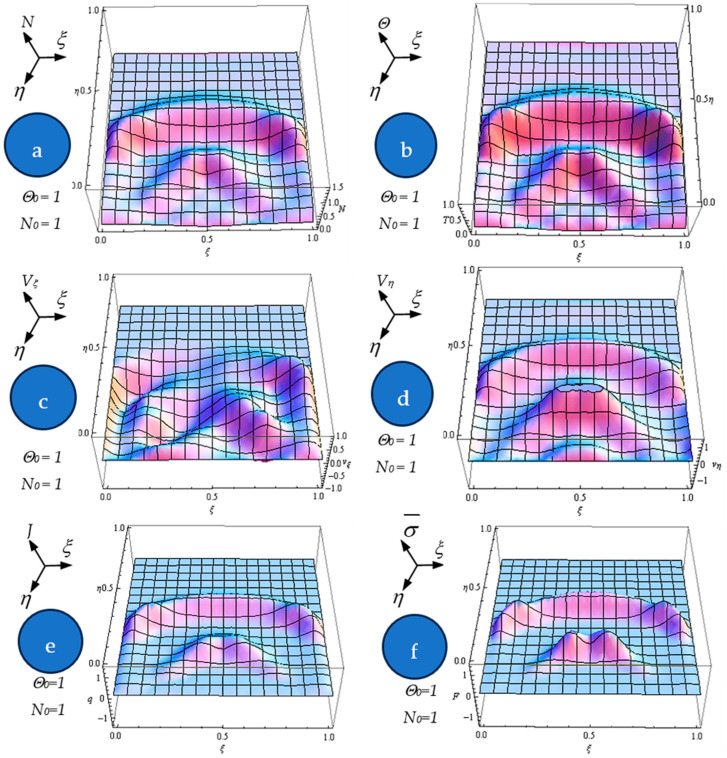
(**a**–**f**) Dependences of the normalized states density *N* (**a**), internal energy Θ (**b**), normalized velocity *V_ξ_* (**c**), normalized velocity *V_η_* (**d**), normalized current density *J* (**e**), and the diagonal component of the normalized internal stress tensor type $\bar{\sigma }$ (**f**) on the normalized spatial coordinates (*ξ*, *η*) at the normalized times *τ* = 0.65 for *N*_0_ = 1 and $\Theta_{0}$ = 1.

**Figure 5:**
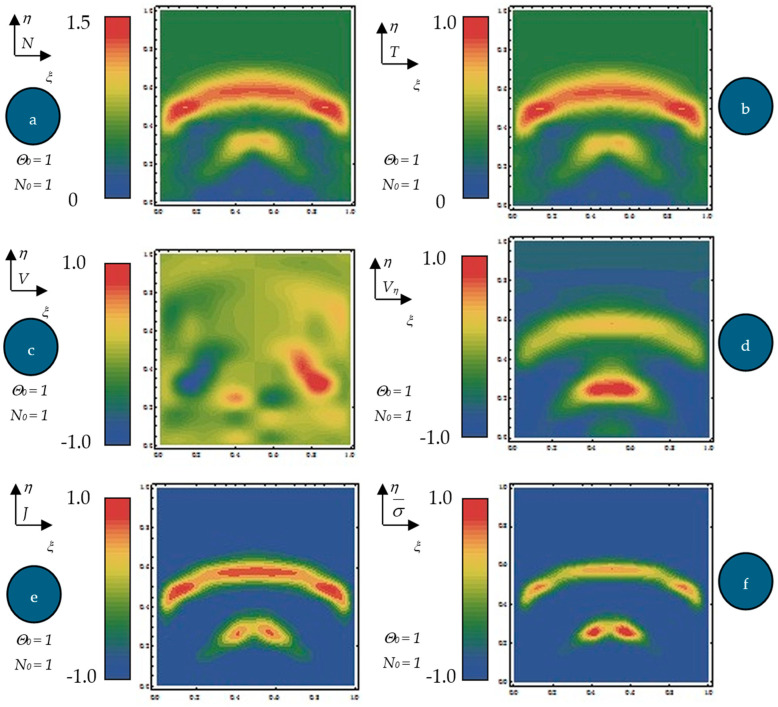
(**a**–**f**) Contour curves of the normalized states density *N* (**a**), normalized internal energy Θ (**b**), normalized velocity *V_ξ_* (**c**), normalized velocity *V_η_* (**d**), normalized current density *J* (**e**), and the diagonal component of the normalized internal stress tensor type $\bar{\sigma }$ (**f**) on the normalized times *τ* = 0.65 for *N*_0_ = 1 and $\Theta_{0}$ = 1.

Equation (61), with the initial conditions (62) and the boundary ones (63), were numerically integrated via finite differences [32]. By means of numerical solutions, in Figure 4a–f, the dependences of the normalized states density $N\left(a\right)$, normalized internal energy per unit volume Θ (b), normalized velocity $V_{\xi }$ (c), normalized velocity $V_{\eta }$ (d), normalized current density $J=N{\left(V_{\xi }^{2}+V_{\eta }^{2}\right)}^{1/2}$, (e) and diagonal component of the normalized internal stress tensor type $\bar{\sigma }=N\left({V_{\xi }}^{2}+V_{\eta }^{2}\right)$ (f) on the normalized spatial coordinates $\left(\xi ,n\right)$ at the normalized times = 0.65 for $N_{0}=1$ and $\Theta_{0}=1$ are plotted. For the same dependences, the contour curves are plotted in Figure 5a–f.

**Figure 6:**
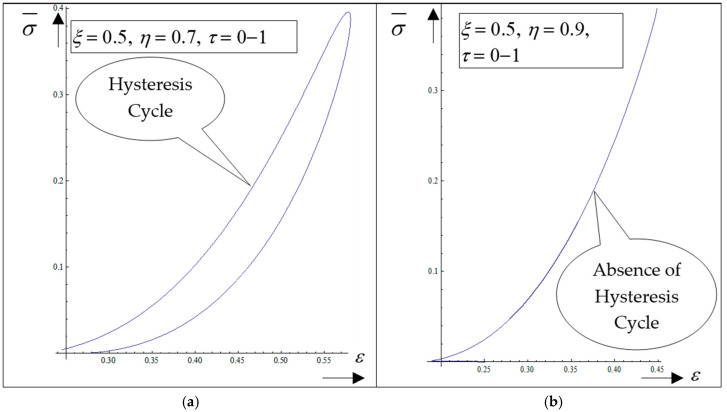
(**a**,**b**) The dependence of the diagonal component of the normalized multifractal stress tensor $\bar{\sigma }$, on the normalized internal energy, Θ for *ξ* = 0.5, *η* = 0.7, *τ* = 0–1 (hysteresis cycle (**a**)) and for *ξ* = 0.5, *η* = 0.9, *τ* = 0–1 (absence of hysteresis cycle (**b**)).

The following results are obtained: (i) The generation of structures in a complex fluid by means of solitons packet solutions [33] (see the peaks from Figure 4a–f and pronounced contours from Figure 5a–f); (ii) the normalized velocity *V_ξ_* (which is normal to the “complex fluid streamline”) is symmetric with respect to the symmetry axis of the space–time Gaussian, while vertices are induced at the periphery of the structures of the normalized velocity *V_η_* (which is along the “complex fluid streamline”); (iii) the coupling of polymer-drug system (as a complex fluid) dynamics at a differentiable scale with the ones at a nondifferentiable scale are performed through the multifractal stress tensor. As a result, the complex fluid entity acquires additional kinetic energy (induced by nondifferentiability) that allows jumps from its own “stream line” to another; (iv) eliminating the time between the diagonal component of the normalized multifractal stress tensor and normalized internal energy per unit volume for various given positions, hysteresis-type effects can be obtained through numerical simulations. For the same *ξ*, such a tendency is more emphasized for the small *η* (Figure 6a—hysteresis cycle), while for the larger *η*, it vanishes (Figure 6b—absence of the hysteresis cycle).

Therefore, in this framework, the presence of the hysteresis cycle bestows memory on the polymer-drug system. This is, in our opinion, the basis of the programmable nature of the polymer-drug system.

## 5. Conclusions

The main conclusions of the present paper are the following:

- A multifractal state density conservation law is employed, utilizing the multifractal motion theory;
- This law is particularized for the case of transition from multifractal to nonmultifractal curves, which, in our opinion, corresponds to the polymer-drug release processes;
- By a particular change in the state density variable in space–time coordinates, a multifractal diffusion-type law is obtained;
- In such a situation, the general solution is found, and it is shown that this solution can describe the cyclic drug release dynamics until the polymer matrix is fully degraded;
- For a well-specified time scale, the release rate is shown to depend both on the velocity associated with the multifractal–nonmultifractal transition and on the diffusion rate;
- In such a context, after each release process, the polymer-drug system repeats the same cycle at a different resolution scale until the drug concentration reaches zero and the polymer network starts to degrade. From this point of view, we can assert that the polymer-drug system exhibits a programmable nature;
- In our opinion, the programmable nature of the polymer-drug system mentioned above is explicated not only by its complex part but also by its scale resolution dependence;
- The temporal release patterns of the channel and cell type, respectively, are emphasized on the basis of a differential geometry induced by a temporal homographic transformation;
- The presence of the hysteresis cycle in polymer-drug systems bestows memory on these systems. This is, in our opinion, the basis of the programmable nature of polymer-drug systems;
- Controlled drug delivery via complex polymer-drug systems and the use of their specific characteristics is a fundamental direction in modern medicine. These technologies, based on advanced scientific principles, make it possible to increase therapeutic efficacy, reduce adverse effects, and tailor treatments to individual patient needs. With continued advances in the field, these systems have the potential to redefine standards in drug delivery, offering personalized and environmentally friendly solutions to health challenges;
- Integrating classical release models with insights from fractal theory can significantly enhance our understanding and prediction of drug release from polymer matrices. Conventional models like Higuchi, Korsmeyer–Peppas, and Weibull remain valuable for their simplicity and have historically enabled researchers to classify and compare release profiles. Yet, their simplifying assumptions limit their predictive accuracy in real-world situations that involve heterogeneous polymer architectures and concurrent processes. Fractal-based approaches address these limitations by capturing the scale-dependent and irregular nature of drug release phenomena.

## Data Availability

All the data are presented in the manuscript.
